# Nephrotic syndrome in the elderly: epidemiological aspects, clinical data, and renal biopsy findings

**DOI:** 10.1590/1414-431X2022e11861

**Published:** 2022-02-28

**Authors:** L.R. Soares, J.M.S. Pantoja, L.B. Jorge, L. Yu, L.B. Cavalcante, D.M.A.C. Malheiros, V. Woronik, C.B. Dias

**Affiliations:** 1 Divisão de Nefrologia, Hospital das Clínicas, Faculdade de Medicina, Universidade de São Paulo, São Paulo, SP, Brasil; 2 Divisão de Anatomia Patológica, Hospital das Clínicas, Faculdade de Medicina, Universidade de São Paulo, São Paulo, SP, Brasil; 3 Laboratório de Fisiopatologia Renal, Faculdade de Medicina, Universidade de São Paulo, São Paulo, SP, Brasil; 4 Departamento de Patologia, Faculdade de Medicina, Universidade de São Paulo, São Paulo, SP, Brasil

**Keywords:** Nephrotic syndrome, Tubulointerstitial fibrosis, Percutaneous renal biopsy, Elderly patients, Epidemiology, Membranous nephropathy

## Abstract

Nephrotic syndrome is the most common clinical presentation of glomerular disease in elderly patients, and renal biopsy is an important diagnostic resource. The aim of this study was to describe nephrotic syndrome among elderly patients in Brazil, focusing on tubulointerstitial and vascular involvement. This was a retrospective study of patients over 65 years of age with nephrotic syndrome who underwent renal biopsy between January 2012 and December 2019. Of the 123 renal biopsies that occurred during the study period, 44 (35.8%) were performed for the investigation of nephrotic syndrome. Among those 44 cases, the main etiologies were membranous nephropathy in 13 cases (29.5%), amyloidosis in ten (22.7%), non-collapsing focal segmental glomerulosclerosis (FSGS) in four (9.1%), and collapsing FSGS in four (9.1%). Patients with minimal change disease (MCD) had the lowest degree of interstitial fibrosis compared with the other glomerulopathies, and histological signs of acute tubular necrosis (ATN) were less common among those with amyloidosis than among those with membranous nephropathy, FSGS, or MCD (P=0.0077). Of the patients with ATN, the frequency of acute kidney injury (AKI) was highest in those with MCD (P<0.001). All patients had some degree of vascular involvement, regardless of the type of glomerulopathy. In conclusion, the second most common cause of nephrotic syndrome in this population was amyloidosis, and acute interstitial tubule involvement was more marked in MCD. Vascular involvement is something that cannot be dissociated from the age of the patient and is not only due to the underlying glomerulopathy.

## Introduction

The aging of the world population, especially in high- and middle-income countries, has prompted discussions regarding renal biopsy for diagnosis of glomerular disease and immunosuppressive therapy in the elderly. In a retrospective study of 434 renal biopsies conducted over a period of approximately 8 years in Shanghai, China (1), 72 (16.6%) were in individuals who were 60 years of age or older. Of the 72 patients, 45 (62.5%) had nephrotic syndrome, which was attributed to membranous nephropathy in 24 (53.3%) cases, minimal change disease (MCD) in 7 (15.6%), focal segmental glomerulosclerosis (FSGS) in 5 (11.1%), and immunoglobulin A (IgA) nephropathy in 2 (4.4%).

In a study of biopsies performed in Japan (2), the main indication for renal biopsy in the elderly (60-79 years of age) and very elderly (≥80 years of age) was nephrotic syndrome. The main etiologies in both groups were, in descending order, membranous nephropathy, MCD, diabetic nephropathy, amyloidosis, and FSGS. However, in a study of patients ≥80 years of age in the United States (3), the main indication for renal biopsy was acute kidney injury (AKI), followed by chronic kidney disease (CKD), nephrotic syndrome, and proteinuria. Among the patients with nephrotic syndrome in that study, the main etiology was membranous nephropathy, followed by amyloidosis and MCD. It is notable that MCD and amyloidosis occupied different positions in those two studies in terms of their prevalence among elderly individuals.

The primary objective of this study was to describe the prevalence and etiology of nephrotic syndrome among elderly patients undergoing renal biopsy in Brazil. A secondary objective was to evaluate aspects of tubulointerstitial and vascular involvement in such patients.

## Material and Methods

This was a retrospective study of patients over 65 years of age who underwent renal biopsy between January 2012 and December 2019 in the Nephrology Department of the Hospital das Clínicas, in the city of São Paulo, Brazil. We evaluated only patients who presented with nephrotic syndrome as defined by edema, proteinuria ≥3.5 g/day, and serum albumin <3.2 g/dL. The study was approved by the Research Ethics Committee of the Hospital das Clínicas. Because of the retrospective nature of the study, the requirement for informed consent was waived.

### Study criteria

We applied the following inclusion criteria: being ≥65 years of age, having undergone renal biopsy for the investigation of nephrotic syndrome, and having undergone a renal biopsy in which the specimen collected contained at least 6 glomeruli (for light microscopy and immunofluorescence). Patients for whom the medical records were incomplete were excluded.

### Variables analyzed at diagnosis

Clinical data such as age, sex, and history of hypertension were collected at diagnosis, as were biochemical data such as hemoglobin, serum creatinine, creatinine clearance (calculated by the CKD Epidemiology equation), serum albumin, 24-h proteinuria or protein/urinary creatinine ratio, and hematuria, as defined by the presence of more than 3 red cells per field confirmed in two samples. From the renal biopsy report, we collected data on the diagnosis of the glomerular disease, the proportional distribution of fibrosis, the presence or absence of acute tubular necrosis (ATN), and any type of vascular involvement. We defined ATN as any degree of necrosis and degenerative or regenerative alterations in the tubular epithelium. Electron microscopy was used only in cases in which light microscopy and immunofluorescence yielded inconclusive results. Patients with a histological diagnosis of ATN who also developed AKI, defined as a ≥50% increase in serum creatinine, were evaluated separately from those who did not.

### Variables analyzed at the end of follow-up

At the end of the follow-up period, the cases of nephrotic syndrome were categorized as being in complete remission, partial remission, or non-responsive. Complete remission was defined as a serum creatinine level that was stable or lower than baseline and proteinuria <0.3 g/day, partial remission was defined as a serum creatinine level ≤25% higher than baseline and proteinuria of 3.0-3.5 g/day, and non-responsive was defined as not meeting any of those criteria.

### Statistical analysis

The Kolmogorov Smirnov test was used, and numerical variables are reported as means±SD if they had a Gaussian distribution or as median and interquartile range (IQR) if they had a non-Gaussian distribution. Categorical variables are reported as absolute and relative frequencies. Numerical variables among the various glomerulopathies were compared with one-way analysis of variance or the Kruskal-Wallis test, as appropriate. Categorical variables were compared with chi-square tests. Values of P<0.05 were considered statistically significant.

## Results

Of a total of 1192 renal biopsies performed during the study period, 123 (10.3%) were in patients ≥65 years of age. Of the 123 cases, the main indication was nephrotic syndrome in 44 (35.7%), followed by rapidly progressive glomerulonephritis in 23 (18.70%). The mean age of the 44 patients with nephrotic syndrome, which was the focus of our study, was 70.25±4.57 years, only two patients (4.5%) were ≥80 years of age, and 26 (59.1%) of the patients were men. At diagnosis, mean serum creatinine was 1.98±1.50 mg/dL, mean hemoglobin was 11.22±1.39 g/dL, mean serum albumin was 2.00±0.41 g/dL, hematuria was seen in 26 cases (59.1%), and mean proteinuria was 8.20±4.06 g/day.

**Figure 1:**
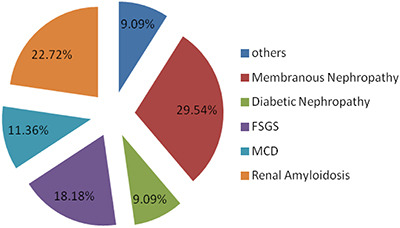
Distribution of the main histological diagnoses from renal biopsies in patients over 65 years of age with nephrotic syndrome. FSGS: focal segmental glomerulosclerosis; MCD: minimal change disease.

Figure 1 shows the distribution of the histological diagnoses. Among the 44 renal biopsies evaluated, the most common etiology was membranous nephropathy, which was reported in 13 cases (29.5%), followed by renal amyloidosis in ten (22.7%), non-collapsing FSGS in four (9.1%), collapsing FSGS in four (9.1%), MCD in five (11.4%), diabetic nephropathy in four (9.1%), lupus nephritis in one (2.3%), fibrillary glomerulonephritis in one (2.3%), membranoproliferative glomerulonephritis in one (2.3%), and IgA nephropathy in one (2.3%).

**Figure 2:**
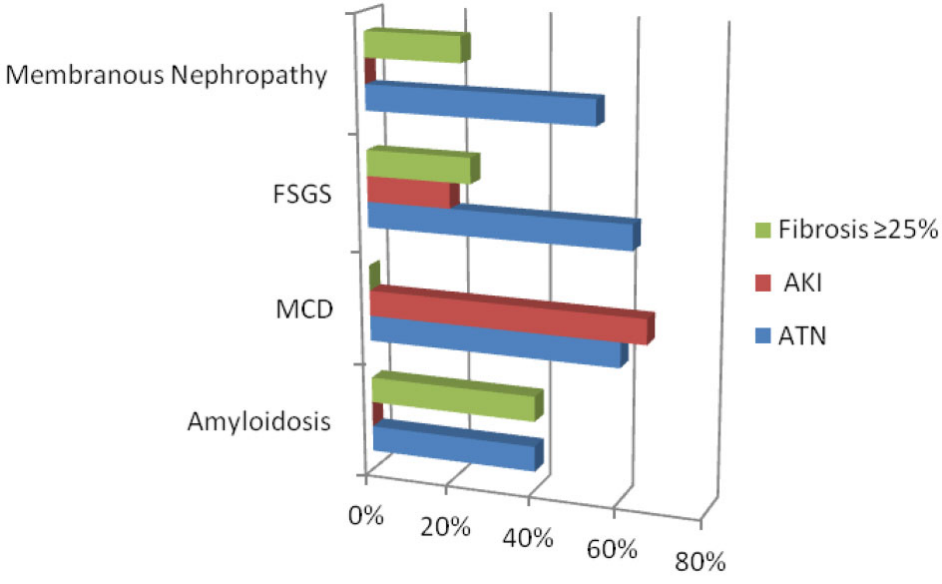
Data on tubulointerstitial involvement and acute kidney injury (AKI) among patients over 65 years of age with nephrotic syndrome by etiology. FSGS: focal segmental glomerulosclerosis; MCD: minimal change disease; ATN: acute tubular necrosis.

At diagnosis, there were no significant differences among the main glomerulopathies in terms of patient age, history of hypertension, serum creatinine, hemoglobin, serum albumin, or proteinuria (Table 1). However, FSGS and MCD were more prevalent in men. In addition, the prevalence of hematuria was lowest among patients diagnosed with amyloidosis or MCD. As expected, the degree of interstitial fibrosis was also lowest among patients with MCD (Table 2). In fact, none of the patients with MCD had fibrosis occupying ≥25% of the parenchyma (P<0.001). In contrast, 25, 23, and 40% of the patients with FSGS, membranous nephropathy, and amyloidosis, respectively, had fibrosis occupying ≥25% of the parenchyma. In the histological evaluation, 100% of the patients were found to have some degree of vascular involvement, regardless of the type of glomerulopathy. One common finding was hypertensive nephrosclerosis, although that was not observed in patients with amyloidosis, all of whom had amyloid deposits in their blood vessels. As illustrated in Figure 2, histological signs of ATN were observed in 40.0% of the patients with amyloidosis compared with 54.0% of those with membranous nephropathy, 62.5% of those with FSGS, and 60.0% of those with MCD (P=0.0077). However, among patients with signs of ATN, the prevalence of confirmed AKI was higher in those with MCD than in those with membranous nephropathy, amyloidosis, and FSGS (66 *vs* 0, 0, and 20%, respectively; P<0.001).

**Table 1:** Demographic and clinical characteristics of patients over 65 years of age with nephrotic syndrome by etiology.

Characteristic	Membranous nephropathy (n=13)	Amyloidosis (n=10)	FSGS (n=8)	MCD (n=5)	P-value
Age (years), mean±SD	70.62±4.40	71.50±4.27	68.78±4.05	71.80±8.07	0.60
Gender (female/male), n	6/7	6/4	1/7	1/4	<0.0001
History of hypertension, n (%)	6 (45.4)	6 (60.0)	4 (50.0)	3 (60.0)	0.17
Hemoglobin (g/dL), mean±SD	11.41±1.44	10.68±1.51	11.21±1.79	11.98±0.99	0.42
Creatinine (mg/dL), median (IQR)	1.30 (0.79-2.16)	1.78 (0.94-3.77)	1.93 (1.30-2.84)	1.09 (0.63-1.92)	0.23
Creatinine clearance* (mL/min/1.73 m^2^), median (IQR)	50.81 (25.84-77.96)	34.90 (11.37-60.57)	42.27 (25.05-54.08)	68.41 (34.48-99.08)	0.33
Albumin (g/dL), mean±SD	1.93±0.59	2.03±0.71	2.13±0.44	1.70±0.53	0.55
Hematuria, n (%)	9 (69.2)	3 (30.0)	7 (87.5)	1 (20.0)	<0.0001
Proteinuria (g/day), mean±SD	8.06±4.85	9.60±4.40	8.58±3.55	7.40±4.29	0.77

FSGS: focal segmental glomerulosclerosis; MCD: minimal change disease; IQR: interquartile range. *Calculated with the Chronic Kidney Disease Epidemiology equation. One-way analysis of variance or Kruskal-Wallis test.

**Table 2:** Proportional distribution of interstitial fibrosis (<25% or ≥25% of the parenchyma) among patients over 65 years of age with nephrotic syndrome by etiology.

Fibrosis	Membranous nephropathy (n=13)	Amyloidosis (n=10)	FSGS (n=8)	MCD (n=5)	P-value
<25%, n (%)	10 (76.9)	6 (60.0)	6 (75.0)	5 (100)	<0.0001
≥25%, n (%)	3 (23.1)	4 (40.0)	2 (25.0)	0 (0)	<0.0001

FSGS: focal segmental glomerulosclerosis; MCD: minimal change disease. ANOVA.

Among the ten patients with renal amyloidosis, only one had AA amyloidosis, which was secondary to rheumatoid arthritis. That patient received immunosuppressive treatment with metrotexate and prednisone for 14 months. The nine remaining patients had AL amyloidosis, of whom two were diagnosed with multiple myeloma and one was diagnosed with cast nephropathy.

For the patients with FSGS, the median follow-up period was 58 months (IQR 7-60 months). Of the eight patients, three progressed to dialysis, one achieved complete remission using prednisone, two were non-responders who were referred to conservative treatment of CKD, and two were lost to follow-up. Among the four patients with collapsing FSGS, there was only one in whom the FSGS was considered a possible etiology or trigger for the underlying disease at the time of the renal biopsy, which was lung cancer.

For the patients with membranous nephropathy, the median follow-up period was 60 months (IQR 24-72 months). Of the 13 patients, three achieved complete remission, one achieved partial remission, three remained under conservative treatment for CKD, and six were lost to follow-up.

For the patients with MCD, the median follow-up period was 48 months (IQR 21-48 months). Of the five patients, four achieved complete remission, without CKD or dialysis, and one was lost to follow-up.

Table 3 shows a comparison among FSGS, membranous nephropathy, and MCD. There were no significant differences in terms of follow-up time. However, as expected, outcomes were significantly better for patients with MCD or membranous nephropathy than for those with FSGS.

**Table 3:** Follow-up and outcomes in patients over 65 years of age with nephrotic syndrome by etiology.

Variable	Membranous nephropathy (n=13)	FSGS (n=8)	MCD (n=5)	P-value
Follow-up time (months), median (IQR)	60 (24-72)	58 (7-60)	48 (21-48)	0.38
Dialysis, n (%)	0 (0)	3 (37.5)	0 (0)	0.03
Conservative CKD treatment, n (%)	3 (23.1)	2 (25.0)	0 (0)	0.16
Remission, n (%)	4 (30.8)	1 (12.5)	4 (80.0)	0.03
Lost to follow-up, n (%)	6 (46.2)	2 (25.0)	1 (20.0)	0.45

FSGS: focal segmental glomerulosclerosis; MCD: minimal change disease; IQR: interquartile range; CKD: chronic kidney disease. One-way analysis of variance or Kruskal-Wallis test.

## Discussion

In a previous study involving 77 patients over 60 years of age who underwent renal biopsy between 1990 and 2006 in Brazil (4), nephrotic syndrome was the main indication for biopsy, accounting for 49.3% of the indications, and membranous nephropathy was the main etiology, followed by amyloidosis and FSGS, in equal proportions. In the present study, nephrotic syndrome was also the main indication for renal biopsy among elderly patients, but in a smaller proportion than those reported in other studies (1,4). This does not mean that we are doing fewer biopsies in elderly patients, rather that there is a difference in years of data collection, with the first study evaluating 16 years and our study evaluating 7 years. The lower proportion of indications for renal biopsy due to nephrotic syndrome can be justified only by the identification and indication of other nephrological syndromes in this age group, such as rapidly progressive glomerulonephritis.

In a study of 4170 renal biopsies conducted in the United Kingdom between 2006 and 2015 (5), 11.1% of the biopsies were in patients ≥70 years of age, similar to the 10.3% observed for patients ≥65 years of age in the present study. In patients categorized as very elderly (typically ≥80 years), the indication for renal biopsy changes, with the main indication being AKI and one of the main diagnoses being pauci-immune vasculitis (3,6). This may partially explain why only two of the patients in our sample were ≥80 years of age.

In our analysis of data related to the four main etiologies of nephrotic syndrome at 65 years of age or older, we found that there was a predominance of men among patients with FSGS and among those with MCD, whereas gender distribution was equal among those with membranous nephropathy and among those with amyloidosis. We also found that the frequency of hematuria was higher in patients with membranous nephropathy or FSGS than in those with amyloidosis or MCD, as is seen in clinical practice.

In a multicenter study involving children and adults with or without glomerular disease, who underwent renal biopsy at various hospitals in China (7), the mean age of the adults was 44.1 years. Among the adults with glomerular disease, 66% had mainly chronic tubulointerstitial involvement, which was seen in 76.2% of those with FSGS, 56.3% of those with membranous nephropathy, and 34.9% of those with MCD. The authors also reported that there was vascular involvement in 80% of the adult patients with FSGS, in 70% of those with membranous nephropathy, and in 35% of those with MCD. It is not possible to compare our study with theirs, because the age groups were different and because those authors did not evaluate amyloidosis. Vascular involvement was observed in all of the biopsies in our sample. We also found that the degree of interstitial fibrosis was similar between patients with membranous nephropathy and those with FSGS, whereas it was practically zero among those with MCD. However, it should be kept in mind that extra-glomerular involvement is strongly associated with shorter renal survival in patients with glomerulopathies (7).

Collapsing glomerulopathy was also described in a study involving 41 patients ≥65 years of age in the United States (8). In that study, the etiology was diabetes mellitus in 17% of the cases, atheroembolic renal disease in 7%, and thrombotic microangiopathy in 5%. The authors found that 11 patients (27%) had ATN, 12 (29%) had severe interstitial fibrosis, and 38 (93%) had arteriosclerosis. In the present study only four patients had collapsing glomerulopathy but we found no definitive etiology or trigger, except in the one patient who had lung cancer.

Renal biopsy studies in elderly patients have shown that performing this procedure is of great benefit because, with proper diagnosis, more patients receive immunosuppressive treatment without an increase in infections and with better survival than in elderly patients who did not undergo renal biopsy (9). In our study, three patients with membranous nephopathy and five with FSGS evolved with CKD or hemodialysis. It is not possible to compare these patients with patients who did not undergo renal biopsy, but it is clear that it would be very difficult to establish a differential diagnosis without the renal biopsy, especially between FSGS, MCD, and amyloidosis.

In conclusion, the second most common cause of nephrotic syndrome in this population was amyloidosis, and ATN with confirmed AKI was more marked in MCD. Vascular involvement is something that cannot be dissociated from the age of the patient and is not only due to the underlying glomerulopathy. Membranous nephropathy and MCD have excellent prognoses, whereas FSGS is likely to result in CKD and the need for dialysis. This should motivate us to intensify our efforts in making the etiological diagnosis of nephrotic syndrome in elderly patients. The limitations of this study lie in the fact that it was a retrospective study conducted at a single center. However, our data are quite comparable to those in the literature and can provide important information for our daily practice.

## References

[1] Chen Y, Li P, Cui C, Yuan A, Zhang K, Yu C (2016). Biopsy-proven kidney diseases in the elderly: clinical characteristics, renal histopathological spectrum and prognostic factors. J Int Med Res.

[2] Yokoyama H, Sugiyama H, Sato H, Taguchi T, Nagata M, Matsuo S (2012). Renal disease in the elderly and the very elderly Japanese: analysis of the Japan Renal Biopsy Registry (J-RBR). Clin Exp Nephrol.

[3] Moutzouris DA, Herlitz L, Appel GB, Markowitz GS, Freudenthal B, Radhakrishnan J (2009). Renal biopsy in the very elderly. Clin J Am Soc Nephrol.

[4] de Oliveira CMJ, Costa RS, Vieira OM, Dantas RAS, Moysés M, Romão EA (2010). Renal diseases in the elderly underwent to percutaneous biopsy of native kidneys. J Bras Nefrol.

[5] Navaratnarajah A, Sambasivan K, Cook TH, Pusey C, Roufosse C, Willicombe M (2019). Predicting long-term renal and patient survival by clinicopathological features in elderly patients undergoing a renal biopsy in a UK cohort. Clin Kidney J.

[6] Verde E, Quiroga B, Rivera F, López-Gómez JM (2012). Renal biopsy in very elderly patients: data from the Spanish Registry of Glomerulonephritis. Am J Nephrol.

[7] Dong J, Li Y, Yue S, Liu X, Wang L, Xiong M (2020). The profiles of biopsy-proven renal tubulointerstitial lesions in patients with glomerular disease. Ann Transl Med.

[8] Kukull B, Avasare RS, Smith KD, Houghton DC, Troxell ML, Andeen NK (2019). Collapsing glomerulopathy in older adults. Mod Pathol.

[9] Yoon HE, Shin MJ, Kim YS, Choi BS, Kim BS, Choi YJ (2011). Clinical impact of renal biopsy on outcomes in elderly patients with nephrotic syndrome. Nephron Clin Pract.

